# From spheroids to organoids: next-generation models for CAR-T cell therapy research in solid tumors

**DOI:** 10.3389/fimmu.2025.1626369

**Published:** 2025-07-11

**Authors:** Mégane Jassin, Alix Block, Laury Désiront, Louise Vrancken, Céline Grégoire, Frédéric Baron, Grégory Ehx, Thi Tham Nguyen, Jo Caers

**Affiliations:** 1 Laboratory of Hematology, Interdisciplinary Cluster for Applied Genoproteomics Institute (GIGA) Institute, University of Liege, Liege, Belgium; 2 Department of Hematology, University Hospital of Liege, Liege, Belgium; 3 Walloon Excellence in Life Sciences and Biotechnology (WELBIO) Department, Walloon Excellenxe in Life Research Institute, Wavre, Belgium

**Keywords:** car-t, chimeric antigen receptor T cells, solid tumor, 3D culture, tumor microenvironment, spheroid, organoid, immunotherapy

## Abstract

Chimeric Antigen Receptor T-cell (CAR-T) therapy is a revolutionary immunotherapy involving the genetic modification of T cells to express chimeric receptors targeting specific tumor antigens. Over the past decade, CAR-T therapy has significantly advanced with the development of five generations of CAR-T cells, each introducing modifications to enhance T cell efficacy, persistence, and the ability to overcome immune evasion mechanisms. The manufacturing of CAR-T cells has also evolved, employing techniques such as viral vector transduction or CRISPR-based gene editing, lipid nanoparticle, or transposon mediated approaches, to optimize their function. However, the development of CAR-T therapy for solid tumors faces significant challenges, primarily due to the hostile tumor microenvironment (TME), which traditional two-dimensional (2D) culture systems fail to accurately replicate. This review explores the potential of three-dimensional (3D) culture models, including spheroids and organoids, as tools for studying CAR-T cells in the context of solid tumors. Unlike 2D models, 3D systems offer a more physiologically relevant environment, better mimicking the TME, tumor heterogeneity, and immune interactions which CAR-T cells must encounter. We examine the advantages and limitations of 2D versus 3D models and discuss four key methods for generating spheroids/organoids: direct cell aggregation, scaffold-based, microfluidic, organs-on-chip and bioprinting, and patient-derived organotypic tumor approaches. Moreover, we explore the use of murine models in preclinical CAR-T research, highlighting their role in studying the dynamics of CAR-T cell trafficking, efficacy, and off-target effects. While CAR-T therapy has shown impressive success in some hematological malignancies, there is still a critical need for improved models to study CAR-T efficacy against solid tumors, particularly in relation to the TME. 2D models remain a valuable tool but should be combined with 3D models and *in vivo* murine studies for more accurate clinical outcome predictions. As we advance toward preclinical and clinical applications, ongoing efforts to develop and refine 3D culture systems are essential for overcoming the unique challenges of CAR-T therapy in solid tumors.

## 1 Introduction to CAR-T therapy

### 1.1 CAR-T cells: from concept to clinical result

A pioneering form of immunotherapy has emerged for cancer treatment: Chimeric Antigen Receptor T-cell (CAR-T) therapy. This highly innovative approach involves genetically modifying a patient’s T cells to express chimeric antigen receptors (CAR) on their surface. These receptors are engineered to specifically recognize antigens present on the surface of tumor cells. After modification, CAR-T cells are reinfused into the patient, where they target and eliminate tumor cells. The concept of leveraging T cells to combat cancer was first pioneered by Steven Rosenberg and colleagues in 1988, when they treated metastatic melanoma patients with tumor-infiltrating T lymphocytes (TILs) isolated from the tumor, expanded ex vivo and reintroduced into patients (1). Moreover, lifileucel, a TIL based therapy was approved in 2024 for metastatic melanoma treatment (2). By creating the first generation of CAR-T cells termed « T-bodies », Zelig Eshhar and his colleagues made significant progress in the field in 1993 (3). CAR-T cells demonstrated impressive outcomes in treating hematological malignancies within the past ten years. In 2017, the U.S. Food and Drug Administration (FDA) approved Kymriah (tisagenlecleucel), the first CAR-T treatment for refractory B-cell acute lymphoblastic leukemia (ALL), (4). It was followed by other CAR-T therapies for lymphomas such as TECARTUS (brexucabtagene autoleulcel), (5) for refractory mantle cell lymphoma and ALL, YESCARTA (axicabtagene ciloleucel), (6) for refractory large B-cell (LBCL) and follicular lymphomas, BREYANZI (lisocabtagene maraleucel, 7) for refractory LBCL and chronic lymphocytic leukemia (CLL), and for refractory multiple myeloma (MM) such as ABCMA (idecabtagene vicleucel), (8) and CARVYKTI (ciltacabtagene autoleulcel, 9). Although ongoing efforts to develop CAR-T therapies for solid tumors, and overcome numerous obstacles related to the hostile tumor microenvironment (TME), further progress is needed before these therapies can reach clinical approval.

### 1.2 CAR-T structure

**Figure 1:**
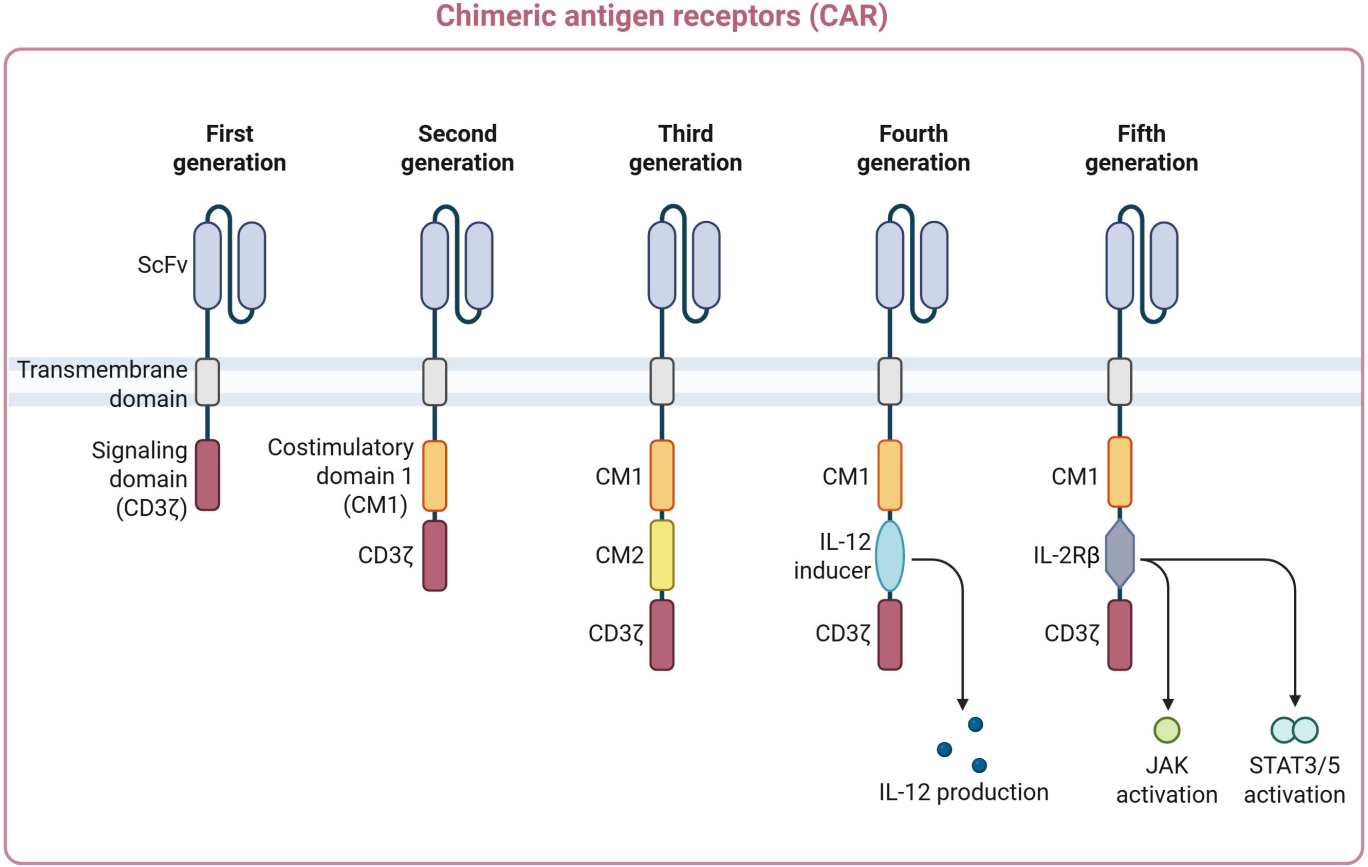
Schematic representation of the five distinct generations of CAR. The first-generation CAR construct consists of an extracellular antigen-recognition domain linked to the intracellular CD3ζ signaling domain. The second-generation contains a co-stimulatory domain (CD28 or 4-1BB) added alongside CD3ζ. The third-generation CAR incorporates more than one co-stimulatory domain. The fourth-generation secretes cytokines upon activation. The fifth-generation CAR incorporates a truncated cytoplasmic domain of the IL-2 receptor β-chain, coupled with a STAT3-binding motif between the co-stimulatory domain and the CD3ζ signaling domain to activate the JAK-STAT. This figure was adapted and generated on Biorender.

Over the years, CAR designs have rapidly improved, resulting in the development of five distinct generations (**Figure 1**). Each generation has added unique characteristics to enhance the efficacy and persistence of CAR-T cells (10, 11). These developments have significantly improved the intrinsic CAR-T cell activity and enhanced the ability to overcome immune evasion of cancer cells and challenges occurring within the hostile TME. While preclinical and early data are promising (12, 13), none of these constructs have yet received FDA approval, excepted CAR-Ts from the second generation. The first-generation CAR construct consists of an extracellular antigen-recognition domain linked to the intracellular CD3ζ signaling domain. In the second-generation construct, a co-stimulatory domain (CD28 or 4-1BB) is added alongside CD3ζ to enhance T cell activation and proliferation. The third-generation CAR construct is similar to the second one but incorporates more than one co-stimulatory domain to enhance CAR T cell activity and persistence. The fourth-generation CAR construct, also called T-cells redirected for universal cytokine killing (TRUCK), is designed to secrete cytokines upon activation to modulate the TME, attract innate immune cells, and improve antitumor efficacy (14). The fifth-generation CAR construct combines a STAT3-binding motif between the co-stimulatory domain and the CD3ζ signaling domain with a truncated cytoplasmic region of the IL-2 receptor β-chain. Without requiring external cytokine support, this design allows for antigen-dependent activation of the JAK-STAT pathway to enhance T cell proliferation and persistence (15).

### 1.3 CAR-T manufacturing

**Figure 2:**
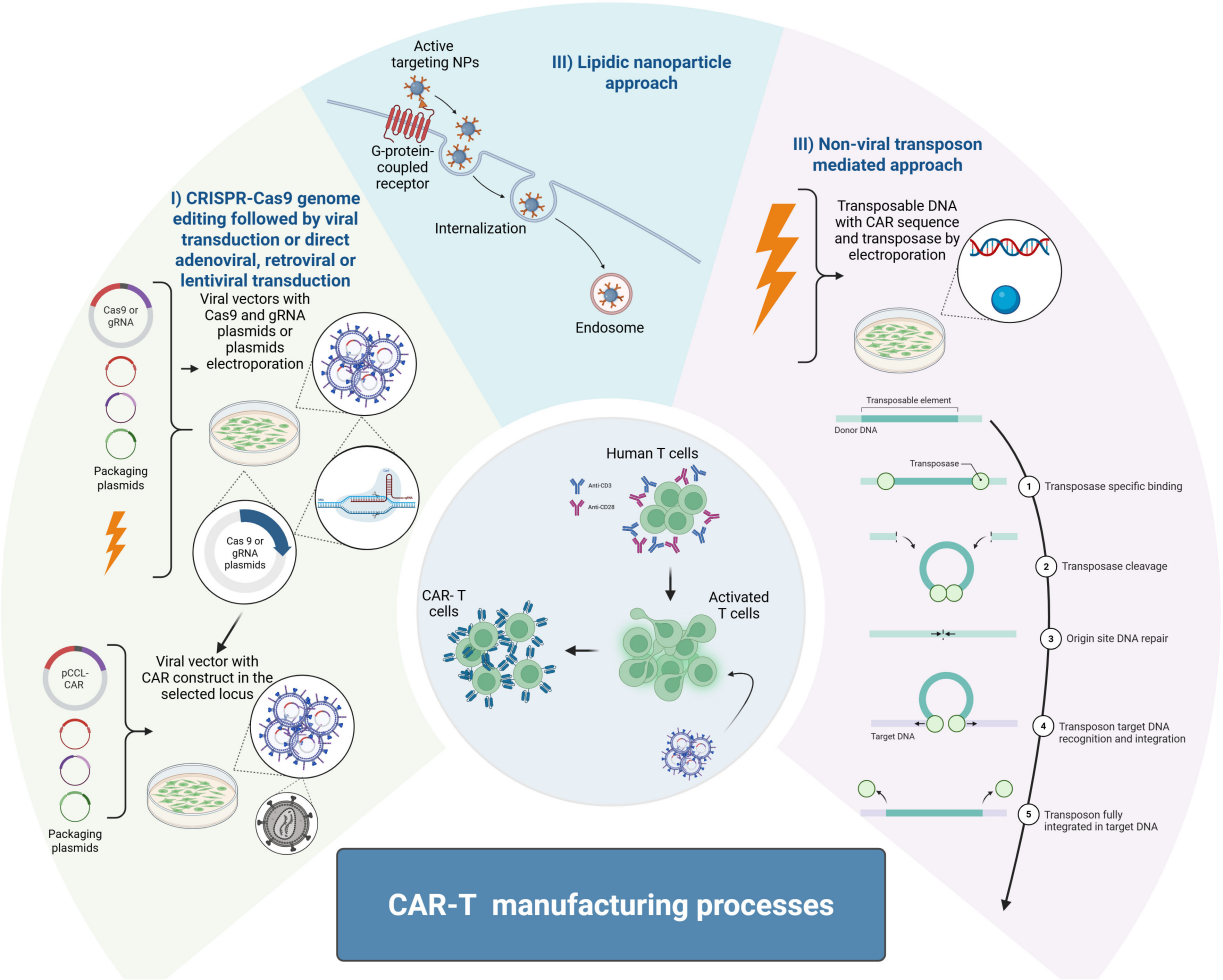
CAR-T manufacturing processes. The first method to generate CAR-T cells is the CRISPR-Cas9 genome editing followed by viral transduction or the direct adenoviral, retroviral or lentiviral transduction of the CAR sequence (I). The second method includes the CAR construct delivered using lipid nanoparticles (II). The third method involves mRNA electroporation transposon-mediated approaches to integrate the CAR sequence into T cells (III). This figure was generated on Biorender.

In addition to design improvement, CAR-T manufacturing has been enriched by several innovations such as CRISPR-Cas9 gene editing to remove immune checkpoint inhibitors such as LAG-3, PD-1 or TIM-3 and enhance resistance to exhaustion and antitumor efficacy (16–18), adenoviral transduction providing efficient gene expression without genome integration and low risk of mutagenesis but offering a short-lived expression (19), retroviral transduction with stable gene integration for a long-term expression but limited to dividing cells with a risk of insertional mutagenesis (19, 20), and lentiviral transduction with stable gene integration for a long-term expression on a broader cell targeting range with lower risk of insertional mutagenesis but with high cost and manufacturing complexity (19). There are also mRNA electroporation, lipidic nanoparticles and transposon-mediated approaches (**Figure 2**) (21, 22). Moreover, CAR-T cells can be manufactured either ex vivo by extracting cells from the patient, engineering them, and reinfusing back or *in vivo* by directly injecting the CAR construct into the patient’s body (23). While *in vivo* CAR-T therapies represent a promising alternative to conventional ex vivo approaches, the first clinical trials have recently begun, and their efficacy and safety remain to be fully established. This is in contrast with second-generation CAR-T therapies produced ex vivo, some of which have already been approved by the FDA. Despite these advances, challenges remain such as immunosuppressive TME, tumor infiltration, antigen downregulation, and CAR-T cell expansion and persistence issues still arise in preclinical and clinical trials. Although results from *in vitro* characterization could differ from those obtained in *in vivo* mouse models and do not always predict outcomes in human clinical trials. Therefore, three-dimensional (3D) co-culture systems appear as attractive intermediate systems between *in vitro* and *in vivo* models to provide more physiologically relevant models for rapid assessment of CAR-T efficacy.

## 2 Advantages and limitations of two-dimensional co-culture systems in CAR-T research

In CAR-T cell research two-dimensional (2D) co-culture systems have served as essential and fundamental tools for preliminary mechanistic investigations and rapid testing of CAR constructs. These systems consist of mixing CAR-T cells directly with tumor cells without any extracellular matrix support, allowing direct cell-cell interactions in a simplified flat 2D environment. While convenient and cost-effective, 2D models remain limited in recapitulating physiological conditions of a complex TME, as they lack crucial immunosuppressive factors, stromal components, and spatial architecture. Thus, results derived from these co-culture systems may be less predictive of *in vivo* responses, limiting their clinical relevance (24). To address these limitations, *in vivo* models such as NOD scid gamma (NSG) and Patient-Derived Xenograft (PDX) provide a TME that better mimics disease conditions such as hypoxia, nutrient gradients and stromal interactions. However, these models still lack host immune interactions due to the use of immunodeficient mice, thereby limiting their ability to fully replicate the immune dynamics observed in patients. Additionally, *in vivo* models remain expensive, time-consuming, and limited by ethical barriers with 3R regulations (Replacement, Reduction and Refinement). Given these challenges, the use of more complex models such as a 3D co-culture and organ-on-chip are in high demand and represent attractive approaches. 3D culture systems incorporate extracellular matrix components, tumor spheroids or organoids, providing a more realistic representation of tumor architecture and cell-cell interactions (25). Organ-on-chip technologies integrate microfluidic systems to recreate tissue-like environments, enabling dynamic perfusion and immune-tumor interactions. These 3D co-culture systems can potentially offer more predictive insights for advancing CAR-T research with conventional 2D and *in vivo* systems (26).

### 2.1 Advantages of 2D models

There are some advantages of using 2D co-culture systems (**Table 1**), such as the ease of use and reproducibility, because these systems are straightforward to establish and follow standardized protocols. Another advantage is the real-time observation because the single-layer arrangement of cells enables direct assessment of CAR-T activity, including cell adhesion, tumor cell lysis and proliferation, through techniques like microscopy and flow cytometry. The absence of a TME enables the targeted study of key parameters such as cytokine production, cytotoxicity, and CAR-T cell proliferation without interference from external factors. Moreover, the 2D cultures can easily integrate a variety of analytical techniques, including ELISA, flow cytometry, lactate dehydrogenase (LDH), or calcium flux assays, facilitating a comprehensive evaluation of CAR-T function. Finally, 2D models remain accessible for widespread use because of their lower cost and minimal specialized equipment required compared to *in vivo* or preclinical research.

**Table 1:** Advantages and limitations of 2D culture assay.

Advantages and limitations of 2D culture assay		
Aspect	Advantages	Limitations
Ease of use and reproducibility	- Straightforward to establish - Standardized protocols ensuring high reproducibility	- Simplistic model may not capture complex interactions
Real-time observation	- Single layer arrangement of cells for direct visualization - Techniques (microscopy, flow cytometry) enable monitoring of CAR-T cell activity, inducing cell adhesion, lysis and proliferation	- Lack of dynamic interaction seen *in vivo* or in more complex systems (3D culture)
Isolation of key parameters	- Isolated study of parameters like cytokine production, cytotoxicity and CAR-T proliferation	- Unable to replicate TME and stromal components
Integration with analytical techniques	- Easily integrates various analytical techniques (e.g. ELISA, flow cytometry, LDH assays)	- Limited capacity to analyze more complex physiological responses such as immune responses
Cost and equipment	- Relatively inexpensive - Minimal specialized equipment required compared to *in vivo* studies	- Cannot replicate systemic immune responses or full physiological context
Realistic tumor microenvironment	- Allows for isolated study of tumor-specific processes (e.g. CAR-T cell activity)	- Lacks realistic gradients of oxygen, nutrients, ECM and interactions between tumor cells and stroma
Tumor cell characteristics	- Use of cell lines to evaluate CAR-T activity	- Tumor cell lines may lose critical aspects of their native phenotype, leading to false results - Use of cell lines instead of primary tumor cells
CAR-T dynamics	- Allows direct interactions between CAR-T and tumor cells	- No physical or biochemical barriers like in a true physiological setting, limiting CAR-T migration and penetration
Immune microenvironment	- Focuses on direct interaction between CAR- and tumor cells	- Cannot simulate immunosuppressive elements of the TME (e.g. CAFs, M2 macrophages, Tregs)

### 2.2 Limitations of 2D models

**Figure 3:**
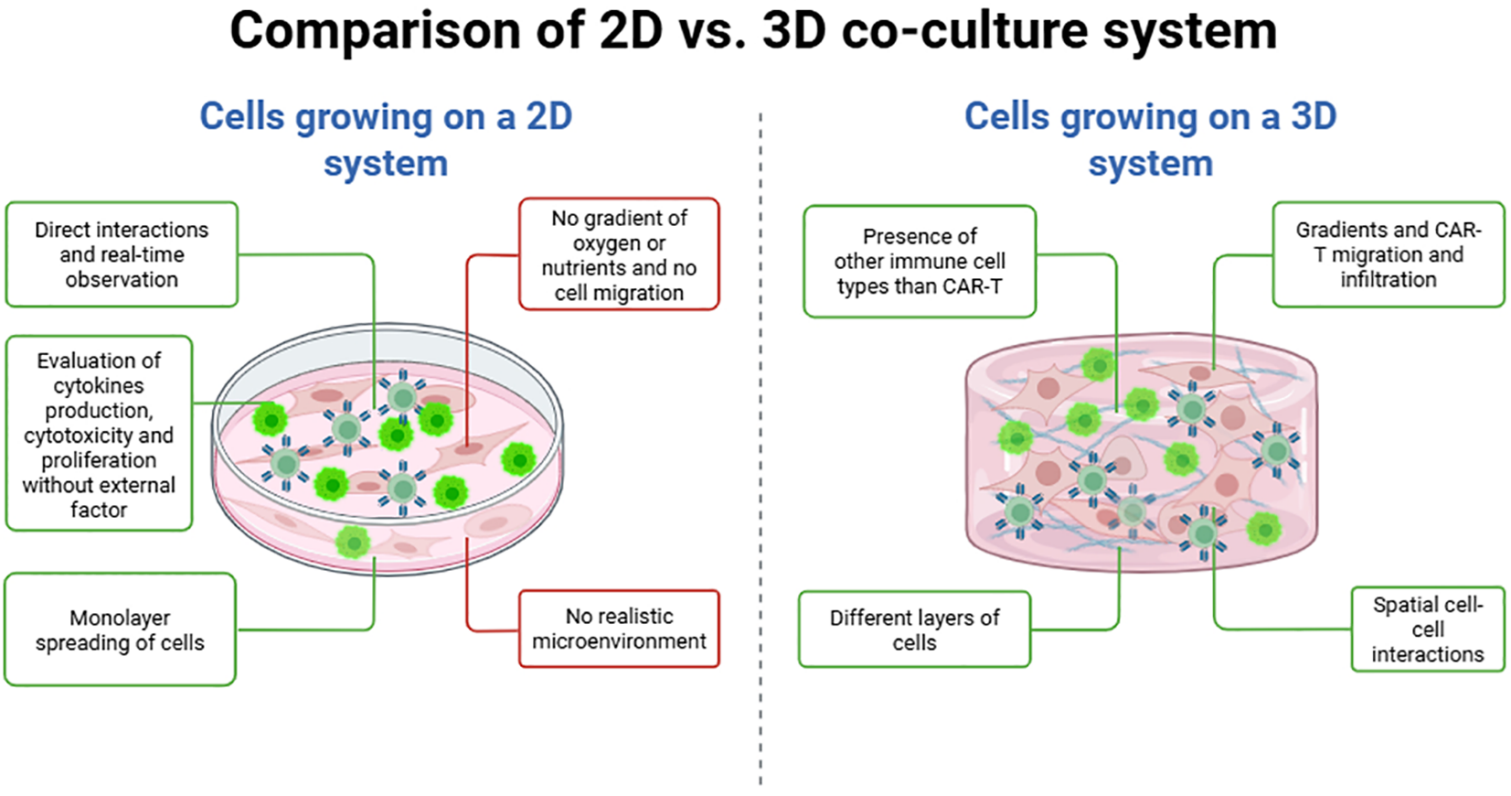
Comparison of 2D vs. 3D co-culture system. In a 2D co-culture system containing a monolayer of tumor cells and fibroblasts, CAR-T cells can interact directly and this could be observed in real-time while evaluating cytokines production, cytotoxicity and proliferation without external factors. However, this system does not represent the microenvironment and the migration of tumor cells or CAR-T cells cannot be followed according to gradient of oxygen or nutrients. In a 3D co-culture system, CAR-T migration, infiltration, cell-cell-interaction and tumor killing can be monitored between the several layers of cells with or without gradients of oxygen and nutrients. This figure was generated on Biorender.

Nevertheless, 2D co-culture systems have several limitations (**Table 1**), such as their inability to recreate a realistic TME. This is due to their failure to replicate oxygen and nutrient gradients as well as the complex interactions between tumor cells and extracellular matrix (ECM). Importantly, the 2D co-culture systems lack the ability to simulate immunosuppressive elements of the TME, such as cancer-associated fibroblasts (CAFs), M2 macrophages, and regulatory T cells (Tregs), which are critical obstacles to solid tumor therapy. The oversimplification can lead to an overestimation of CAR-T efficacy (27). Tumor cells in 2D models use cell lines, naturally expressing the target antigen, which may cause a loss of their native tumor phenotype and lead to false-positive results regarding CAR-T efficacy (28). Moreover, 2D culture seems not suitable for some primary cells, such as primary CLL and MM cells for hematological malignancies or primary pancreatic ductal adenocarcinoma (PDAC) which undergo rapid apoptosis or senescence *in vitro*, and the use of cell lines does not fully recapitulate the biology of the disease. However, cultivating primary cells in 3D models might provide the right microenvironment to support cancer cell survival, making it possible to study CAR-T cell activity for a longer time than in 2D cocultures. Furthermore, in a physiological context, CAR-T cells must overcome physical and biochemical barriers before interacting with tumors cells. This complexity is not adequately represented in 2D models, where CAR-T cells directly interact with tumor cells (29). Thus, 2D systems do not capture dynamics like CAR-T cell migration, penetration into dense tumor tissues, or exhaustion in hypoxic, poorly vascularized environments, and fail to include essential stromal and vascular components, which significantly influence tumor growth, immune evasion, and CAR-T cell functionality (28). Because of their simplicity to use, low cost and analytical compatibility, 2D culture systems remain indispensable for initial CAR-T cell research. However, their inability at accurately replicating the complexities of the TME highlights the necessity to switch to more physiologically relevant models. To fill these gaps, researchers increasingly depend on 3D co-culture systems to provide a transitional step between basic *in vitro* studies and *in vivo* validation. This allows for a more accurate evaluation of CAR-T behavior and efficacy in realistic tumor environments (**Figure 3**).

## 3 Three-dimensional co-culture system: a bridge between 2D and *in vivo* studies

**Figure 4:**
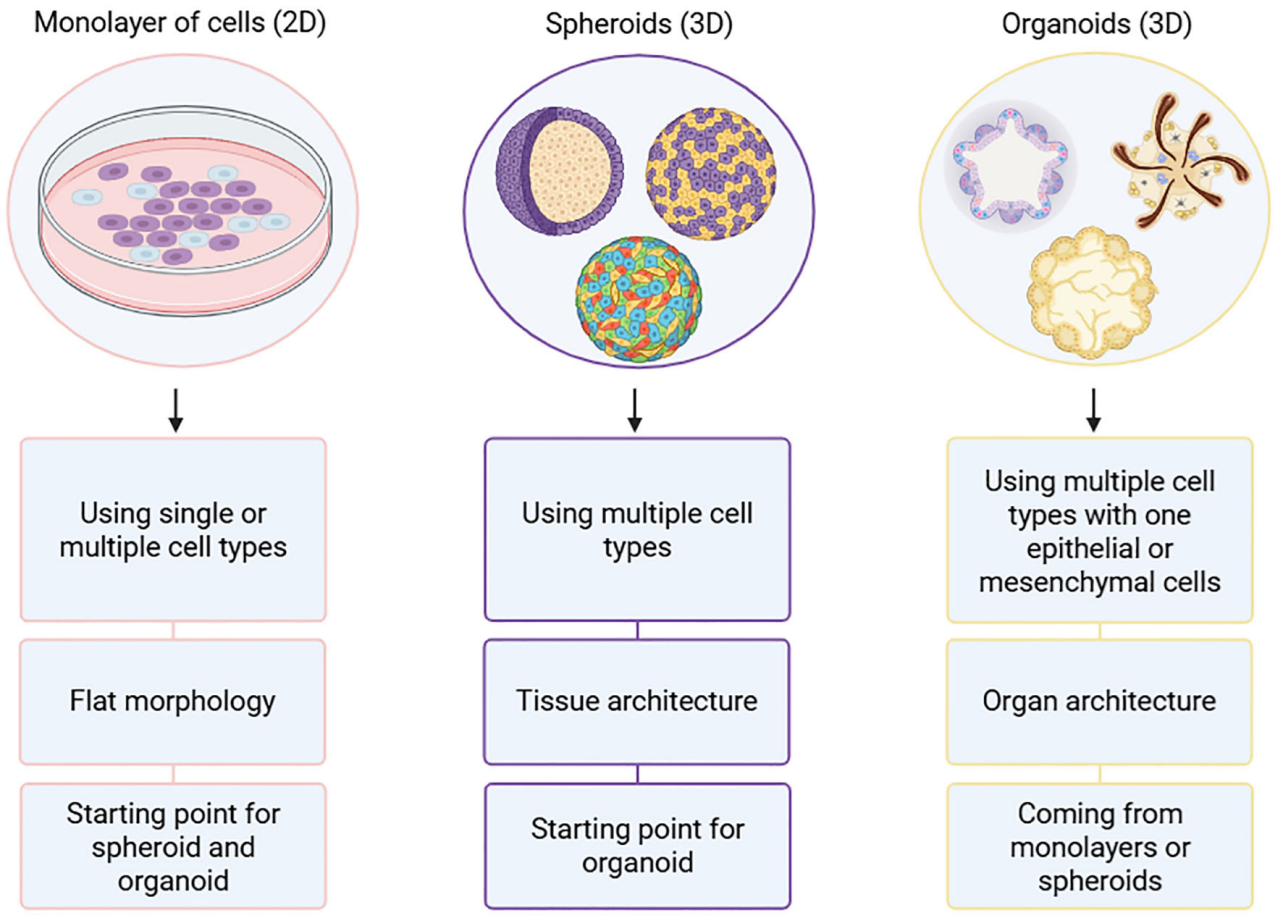
Differences between monolayer 2D cultures, spheroids and organoids. Monolayer cultures consist of single cell type or multiple cell types while spheroids and organoids are composed of multiple cell types. Additionally, organoid structure requires at least epithelial or mesenchymal cells. The architecture complexity increases from monolayers (flat structure) to spheroids (tissue-like aggregates) and organoids (organ-like structures). Furthermore, organoids are composed of monolayers or spheroids composed of monolayers. This figure was generated on Biorender.

The introduction of 3D cell culture systems marked a significant advancement in research, offering a more physiologically relevant model than traditional 2D cultures (**Table 2**). By enabling spatially organized cell interactions, these 3D systems better mimic the natural tissue microenvironment, which is essential for studying complex processes like cancer progression, immune responses, and tissue regeneration. In the context of solid tumors, 3D co-culture systems provide valuable insights into CAR-T cell interactions with the TME, revealing key challenges such as tumor resistance, immune evasion, and infiltration limitations. This approach enhances the evaluation of therapeutic outcomes and helps refine CAR-T cell strategies for improved efficacy in solid tumors (28, 30). In 1956, Ehrmann and Gey replaced the flat-surface cell culture method with the culture of human cell lines in rat tail-isolated collagen to promote cell aggregations in 3D (31). However, the term « spheroid » emerged in 1970 to describe Rheinwald and Green 3D cell culture using human progenitor cells where cells aggregate as a sphere (32). In 1980’s and 1990’s, they further developed *in vitro* cultures from neuroblastomas and lung tissues considered to as the first organoids, a self-organizing structure derived from stem cells, mimicking key features of an organ. Indeed, spheroids are related to tissue architecture whereas organoids are linked to organ architecture because cells spontaneously organize themselves to form a structure similar to the organ they derived from (33). Moreover, organoids can be derived from spheroids (34), (**Figure 4**). Finally, Rheinwald and Green’s further investigated 3D culture and started to cultivate skin organoids from human primary cells (35, 36).

**Table 2:** Comparison between 2D and 3D co-culture systems for CAR-T research.

Criteria	2D co-culture systems	3D co-culture systems
Structure	Monolayer, flat surface	Spatial, multicellular aggregates or matrix-embedded culture
Ease of use	Simple to set up, standardized protocols	Can be a more complex set up, can require specialized techniques
Cost	Low	Low
Reproducibility	High	Moderate, depends on matrix and culture conditions
Real-time observation	Easy (microscopy, flow cytometry)	More difficult due to depth and opacity
Simulation of TME	Poor, lacks extracellular matrix and stromal cells	Good, allows inclusion of stromal cells, ECM, and gradient formation
Immune suppressive components	Absent (e.g., CAFs, Tregs, M2 macrophages)	Can be integrated
CAR-T cell migration/infiltration	Not evaluated, direct contact with tumor cells	Assessed, mimics physical barriers and infiltration
Cytokine-cytotoxicity assessment	Accessible (ELISA, LDH, flow cytometry, etc.)	Possible but more technically demanding
Suitability for primary cells	Often limited, primary cells may not survive	Better, supports viability and function of fragile primary cells
Physiological relevance	Low	High
Use case	Initial screening, functional assays	Intermediate valisation before in vivo testing

## 4 Strategies for culturing spheroids and organoids including CAR-T cells

Three-dimensional culture systems offer more physiologically relevant models for studying CAR-T cells in solid tumors where they face challenges such as immune suppression and poor tissue penetration. Unlike hematological cancers, in solid cancers, CAR-T cells must infiltrate dense extracellular matrices and pass through multiple barriers created by stromal cells and deposited matrix proteins, making *in vitro* 3D models essential for *in vivo* studies or preclinical evaluation. This section highlights strategies to generate spheroid and organoid cultures and integrate CAR-T cells to investigate their efficacy in solid tumor therapy (**Table 3**).

**Table 3:** Strategies to produce spheroids or organoids while studying CAR-T cells in solid tumors.

1. Direct formation		
Cancer models	Target for CAR-T	Studies
Nasopharyngeal carcinoma stem cells	5T4 (trophoblast glyocoprotein)	(42)
Ewing sarcoma	Vascular Endothelial Growth Factor Receptor 2 (VEGFR2)	(43)
Lung cancer, breast cancer, gastric cancer, pancreatic cancer	Mesothelin	(22)
Glioblastoma	Disialoganglioside GD2	(21)
Osteosarcoma	Disialoganglioside GD2	(37)
Epithelium and mesenchymal stromal cells	Human Epidermal Growth Factor Receptor 2 (HER2)	(38)
Breast cancer	Folate Receptor Alpha (FRα)	(44)
Ovarian cancer	Tumor-Associated Glyocprotein 72 (TAG-72)	(45)
Squamous cell carcinoma	Cluster of Differenciation 98 heavy chain (CD98hc)	(39)
Ovarian cancer	Mesothelin	(46)
Canine sarcoma	B7 Homolog 3 (B7-H3 or CD276)	(47)
Lung cancer	Human Epidermal Growth Factor Receptor 2 (HER2) and Human Leukocyte Antigen A*02 (HLA-A*02)	(48)
Epithelial and mesenchymal cells	High-Mannose Glycans	(49)
Breast cancer-associated fibroblasts	Folate Receptor Alpha (FRα)	(40)
Prostate adenocarcinoma and mesenchymal stem cells	Cluster of Differenciation 19 (CD19)	(50)
Chordoma	B7 Homolog 3 (B7-H3 or CD276)	(41)
Glioblastoma	Cluster of Differenciation 44 (CD44)	(51)
Chronic lymphoblastic leukemia	Cluster of Differenciation 19 (CD19)	(52)
2. Use of a matrix		
Cancer models	Target for CAR-T	Studies
Glioblastoma	Epidermal Growth Factor Receptor (EGFR)	(64)
Colorectal cancer	Doublecortin-Like Kinase 1 (DCLK1)	(96)
Breast cancer	Human Epidermal Growth Factor Receptor 2 (HER2)	(54)
Cartilage and chondrocytes	Collagenase II (CII)	(74)
Cholangiocarcinoma	Integrin αvβ6	(55)
Medulloblastoma	B7 Homolog 3 (B7-H3 or CD276)	(65)
Chodroma	B7 Homolog 3 (B7-H3 or CD276)	(56)
Lung cancer	Mesenchymal-Epithelial Transition Factor (MET)	(61)
Breast cancer	Human Epidermal Growth Factor Receptor 2 (HER2)	(66)
Retinoblastoma	Disialoganglioside GD2	(67)
Cholangiocarcinoma	Mucin	(57)
Melanoma	Chondroitin sulfate proteoglycan 4	(68)
Melanoma	Human Epidermal Growth Factor Receptor 2 (HER2)	(58)
Breast cancer	Natural Killer Group 2 Member D (NKG2D)	(69)
Hepatocellular carcinoma	Cluster of Differenciation 39 (CD39)	(62)
Stromal cells	Cluster of Differenciation 19 (CD19)	(63)
Gastric carcinoma	Carcinoembryonic Antigen (CEA)	(70)
Hepatocellular carcinoma	Folate Receptor Alpha (FRα)	(71)
Ovarian cancer	Mesothelin	(59)
Glioblastoma	Disialoganglioside GD2	(73)
Ovarian cancer	Mucin-1	(75)
Breast cancer	Human Epidermal Growth Factor Receptor 2 (HER2)	(60)
Gastric cancer	Human Epidermal Growth Factor Receptor 2 (HER2)	(76)
3. Use of microfluidic, organ-on-chip or bioprinting.		
Cancer models	Target for CAR-T	Studies
Ovarian adenocarcinoma	Mesothelin	(83)
Ovarian cancer	Tumor-Associated Glyocprotein 72 (TAG-72)	(45)
Breast cancer and cancer-associated fibroblasts	Epidermal Growth Factor Receptor (EGFR), Human Epidermal Growth Factor Receptor 2 (HER2) and Natural Killer Group 2 Member D (NKG2D)	(79)
Breast and ovarian carcinoma	Human Epidermal Growth Factor Receptor 2 (HER2)	(81)
Thyroid, kidney, lung, ovary and cancer-associated fibroblasts	Mesothelin	(80)
Colorectal adenocarcinoma	C-X-C Chemokine Receptor Type 3 (CXCR3)	(78)
Breast cancer	Epidermal Growth Factor Receptor (EGFR), Receptor Tyrosine Kinase-Like Orphan Receptor 1 (ROR1), C-C Chemokine Receptor Type 9 (CCR9 or CD199)	(85)
Breast cancer	Human Epidermal Growth Factor Receptor 2 (HER2)	(86)
4. Patient-derived organotypic tumor or tumor slice culture assays.		
Cancer models	Target for CAR-T	Studies
Breast cancer	B7 Homolog 3 (B7-H3 or CD276), Human Epidermal Growth Factor Receptor 2 (HER2), Epidermal Growth Factor Receptor (EGFR), Trophoblast Cell Surface Antigen 2 (TROP2), Epithelial Cell Adhesion Molecule (EpCAM)	(88)
Bladder cancer	B7 Homolog 3 (B7-H3 or CD276)	(89)
Melanoma liver metastases	B7 Homolog 3 (B7-H3 or CD276)	(90)
Pancreatic adenocarcinoma	Mesothelin	(93)
Glioblastoma	Epidermal Growth Factor Receptor (EGFR) and Interleukin 13 Receptor Alpha 2 (IL13Rα2)	(94), NCT05168423
Glioblastoma	Ephrin Type-A Receptor 3 (EphA3)	(91)
Ovarian cancer	Purinergic Receptor P2X (P2X7)	(92)
Renal carcinoma	Carbonic Anhydrase IX (CAIX)	(95)

### 4.1 Cell aggregation

**Figure 5:**
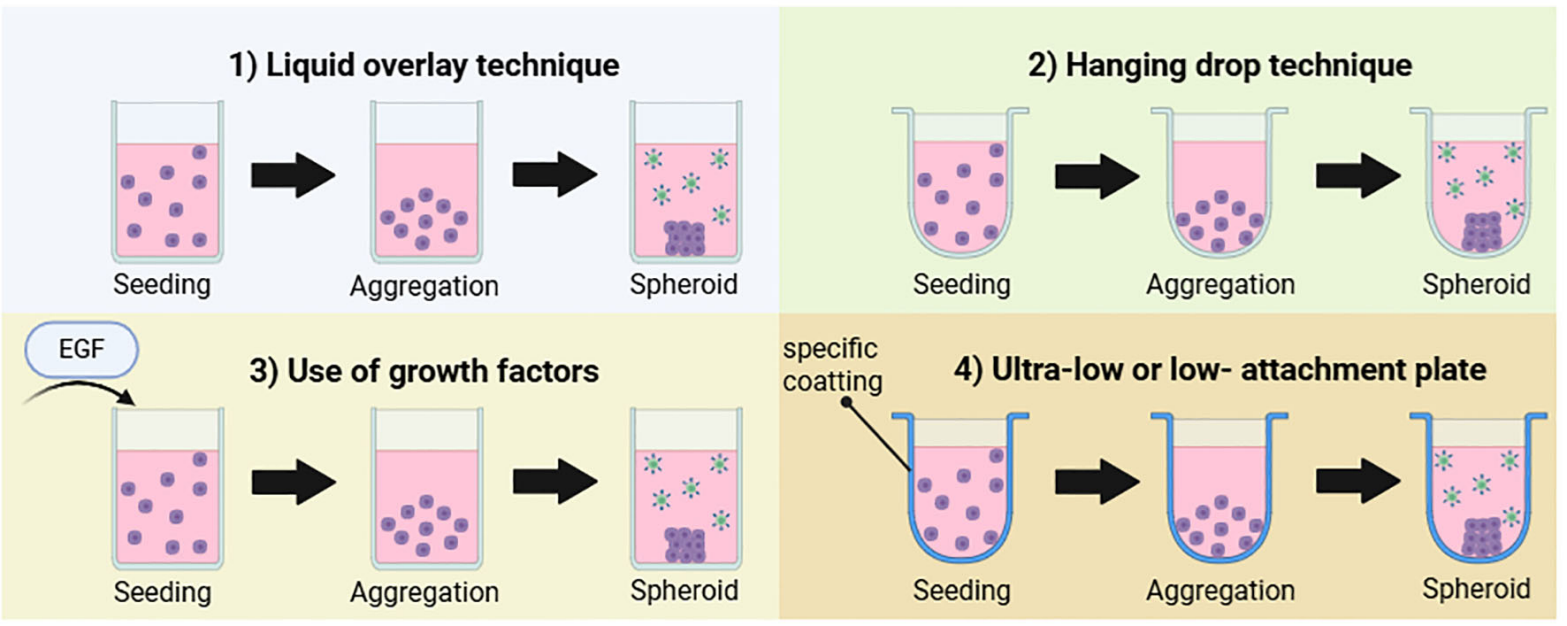
Schematic representation of direct spheroid formation. First method is the liquid overlay technique where cells aggregate together spontaneously to form a spheroid after being seeded. Second method is the hanging drop technique using a U-bottom well to facilitate natural aggregation. Third method is the same as the first one while cells are seeded by growth factors. Fourth method is the use of ultra-low or -low attachment plate with a specific coatting to avoid cell adherence to plastic. This figure was generated on Biorender.

The first method for generating spheroids is direct formation by letting cells aggregate spontaneously together in a flat-bottom plate such as the liquid overlay technique, or in a U-bottom plate such as the hanging drop spheroid (22, 37–41). In these techniques, cell aggregation can be facilitated using low or ultra-low attachment plates coated to prevent cell adherence to the plastic surface. This coating promotes cells to interact with each other, forming small spherical structures (42–51). Additionally, 1% of agarose can be added to the plate bottom to maintain the stemness of cells when necessary, as seen in organoid formation using stem-cell-derived beta cells using plate shaking techniques (37, 38, 52),. Alternatively, direct spheroid formation can be fostered by adding specific molecules and factors to mimic the TME and promote cell growth and aggregation. For instance, for brain cancer and glioblastoma models, epidermal growth factor (EGF), basic fibroblast growth factor (bFGF) or insulin-like growth factor (IGF) are used along with neurobasal medium supplemented with B27 or N2 to replicate brain tumor signaling and support neural cell growth (**Figure 5**; 51, 53),. For CAR-T cell studies, CAR-T cells are subsequently introduced into the established 3D models using different strategies, depending on the research goals. For instance, to study CAR-T cell-mediated cytotoxicity, CAR-T cells can be added directly with tumor cells for co-culture into the spheroid during the initial aggregation phase. Alternatively, to investigate infiltration dynamics, CAR-T cells can be introduced after spheroid formation.

### 4.2 Using matrix and scaffold

**Figure 6:**
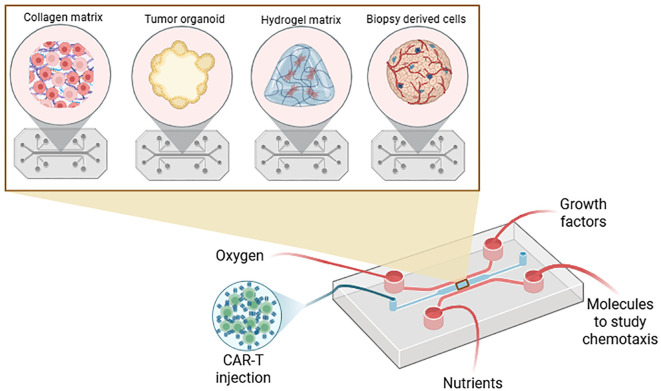
Schematic representation of using matrix coupled with microfluidic, organ-on-chip and bioprinting. Microfluidic device is composed of different channels, one to culture the spheroid or organoid with CAR-T cells while other channels can bring oxygen, nutrients, chemotaxis molecules or growth factors or can mimick blood vessels. Channels can be coupled with filters to remove debris and dead cells. This figure was generated on Biorender.

The second method for producing spheroids or organoids is to use a matrix as a scaffold (**Figure 6**). There are several matrices available such as matrigel (54–63), hydrogel (64–73), and collagen (74–76) from rat or mouse tails. In this model, cells are cultured in the matrix as in 3D space instead of monolayer. Another kind of matrix is the ECM derived from tissues and organs. ECM can provide a more natural microenvironment compared to synthetic hydrogels, such as polyethylene glycol (PEG) or poly-lactic-co-glycolic acid (PLGA), which facilitate the creation of more physiologically and biochemically relevant models for spheroid and organoid formation (54, 60, 66, 69), (73), (75),,– (77). Another way is to produce the spheroid using a low-attachment plate before transferring it into a gel bubble to let it grow in 3D (55, 56),. CAR-T cells can be directly added into pre-formed spheroids or organoids in the matrix or into the culture medium surrounding the spheroid/organoid (65, 67),. To investigate the early interactions with tumor cell aggregates, or CAR-T activation and tumor killing (72), CAR-T cells can be co-cultured before or during the formation of the spheroids/organoids. On the other hand, to evaluate CAR-T infiltration, migration as well as killing tumor cells, CAR-T cells can also be added after the spheroid/organoid formation as a well-established tumor structure. This method mimics the conditions of treating an existing tumor rather than targeting cells in the early stages of aggregation.

### 4.3 Advanced techniques: microfluidic, organs-on-chip, or bioprinting technologies

The third method for creating spheroids and organoids involves microfluidic, organs-on-chip, or bioprinting technologies (**Figure 6**). Several devices and chips have been engineered to deliver oxygen or nutrients, regulate and study chemotaxis (78, 79), mimic blood vessels and vascularization (80), remove dead cells and deliver CAR-T cells using microdroplets (81) or microhydrogels (45, 82, 83),. In these approaches, spheroids, organoids, or biopsy-derived cells are cultured on a matrix and connected to microfluidic systems (78, 84). Similar to microfluidic systems, organ-on-chip provides precise regulation of biochemical and physical microenvironments, enabling accurate modeling of tumor microenvironment complexity and CAR-T cell behavior. Additionally, organ-on-chip systems simulate blood flow to allow the investigation of CAR-T cell infiltration and interactions with endothelial barriers, which is critical for studying solid tumors (82, 84–86). Using multiple channels, these devices allow simultaneous testing on various conditions, such as cytokine gradients or different CAR construct injections (86). Most recently, bioprinting has emerged as a cutting-edge technology offering precise tissue construction (87). Thus, these technologies facilitate the 3D dynamic studies of CAR-T cell migration, proliferation, and cytotoxicity under well-controlled parameters, giving kinetic results that static cultures cannot achieve.

### 4.4 Patient-derived organotypic tumor or tumor slice culture assays

**Figure 7:**
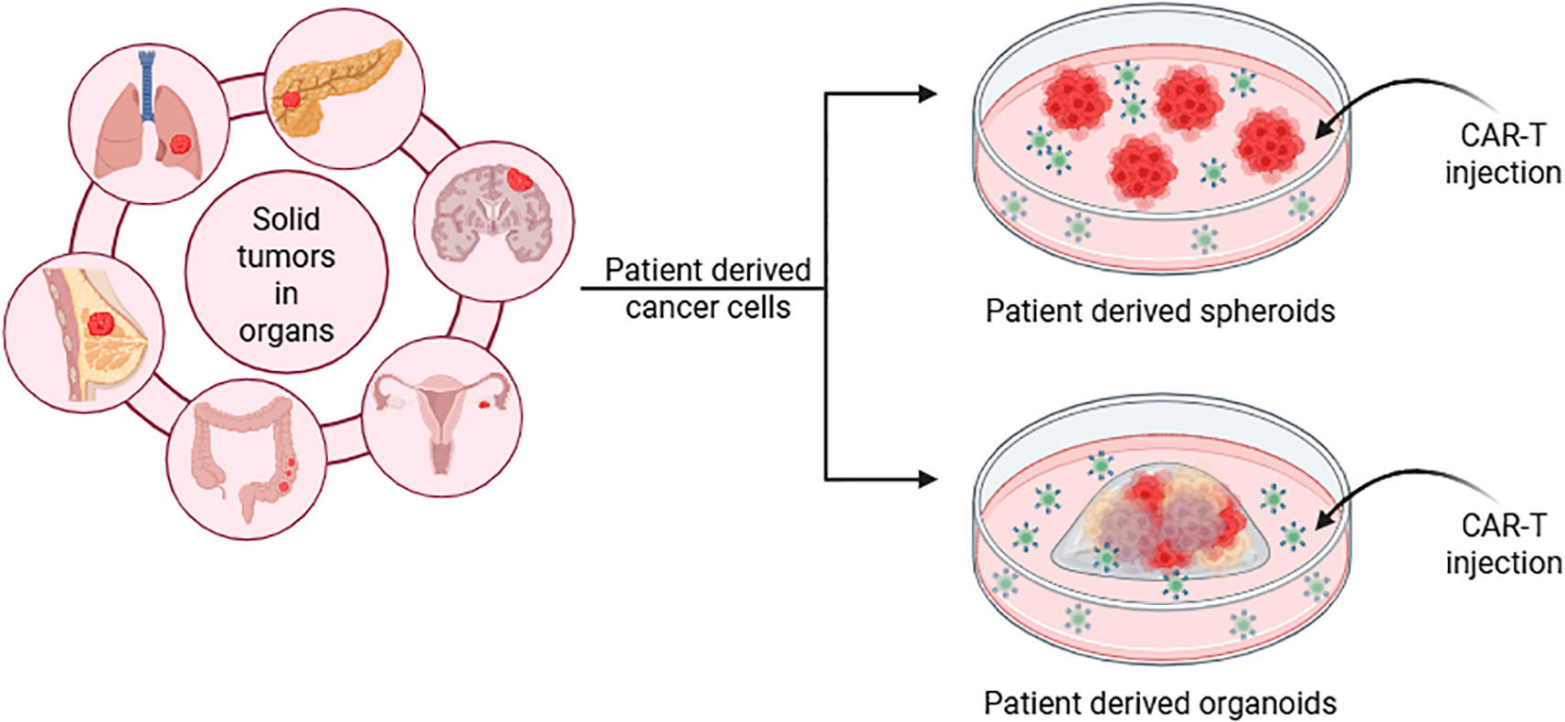
Schematic representation of patient-derived organotypic tumor preparation. Solid tumors are extracted from human patient organ biopsies and cultured on a matrix. Extracted tumor can be sliced depending on their size and can be cultured as organoids or spheroids before injecting CAR-T cells. This figure was generated on Biorender.

The fourth method is patient-derived organotypic tumor or tumor slice culture assays (**Figure 7**). This technique is related to ex vivo studies, where human tumors are extracted from the patient and cultured on a matrix (21, 88–94). Depending on the size of the tumor and the microenvironment components extracted, the tumor can be cultured whole or sliced into thin sections (93). These cultures can also be connected to a microfluidic system to supply medium, and nutrients or to facilitate CAR-T cell injection (95). Alternatively, CAR-T cells can be introduced using similar methods to those described in the second method of organoid manufacturing.

### 4.5 Methods to study CAR-T cells in a 3D co-culture system

As with 2D analysis, several methods can be used to study CAR-T cell behavior in a 3D co-culture system. Indeed, confocal microscopy and live-cell imaging are used to track CAR-T activity such as the infiltration into the spheroid/organoid, the spatial distribution of tumor and CAR-T cells, CAR-T migration, and cytotoxicity. To track CAR-T and tumor cells in the spheroid/organoid, cells are labelled with a fluorescent molecule or a reporter system. Flow cytometry can also be used after the dissociation of the co-culture system into single-cell suspensions to further analyze CAR-T cell activity, cytotoxicity, persistence, expansion and proliferation, differentiation phenotype, and exhaustion. Coupled with flow cytometry, ELISAs can be employed to analyze release cytokine production for CAR-T cells such as TFN-α, IFNγ, and IL-2. Viability assays using LDH release assays or live-dead staining can be used to quantify tumor cell damage or death before and after CAR-T treatment. Finally, compared to 2D co-culture systems, histological analysis can be applied to fixed spheroids/organoids using immunohistochemistry (IHC) or immunofluorescence to detect tumor killing or apoptosis, CAR-T cell infiltration and phenotype (97).

## 5 From mouse to clinic: murine models driving preclinical progress

*In vivo* research provides key insights into the physiological complexities of cancer treatments and acts as an essential link between *in vitro* studies and human clinical trials. For CAR-T cell research, murine models are the most commonly used, enabling researchers to assess the efficacy, safety, and pharmacokinetics of treatments in a living organism, and dynamics, including their migration, proliferation, and cytotoxic effects in the context of a systemic environment where cancer may be disseminated. CAR-T cell interactions that are missing in 3D culture systems are typically represented in these models, including interactions with the broader biological system, such as blood vessels, and distant tissues to evaluate potential off-target effects and toxicity in non-tumor tissues. *In vivo* studies allow researchers to investigate systemic interactions such as immune responses, biodistribution, and off-target effects on healthy tissues in the context of a fully functional organism. However, studying immune-mediated processes is limited by the use of immunodeficient mice. Moreover, *in vivo* experiments remain expensive, time-consuming, resource-intensive, and subject to ethical restrictions. Despite these challenges, *in vivo* models offer a global insight of therapeutic effects in a living dynamic biological organism. Depending on different cancer models, tumor cells can be injected subcutaneously using matrigel or not [e.g. melanoma (98), breast cancer (99), prostate cancer (100), rhabdomyosarcoma (101), mesothelin (102)], intravenously in the blood-stream [e.g. hematological malignancies (103–105)], intraperitoneally [e.g. ovarian cancer (106)] or orthotopically in the organ of origin [e.g. breast cancer (99), glioblastoma (91), lung cancer (107), glioma (108)]. Furthermore, murine models are also used to evaluate procedures commonly used in CAR-T therapies such as isolation of T cells, their genetical engineering to express the CAR, and their expansion before reinfusion. Moreover, CAR-T cells can be administered either directly into the tumor to study local effects or intravenously to observe their migration, tumor-homing behavior, and systemic activity. Compared to preclinical or clinical trials on human patients, there are some limitations of murine models such as the use of murine cells, or xenografted human cells injected in immunocompromised mice lacking some immune cells, and the issue of lack of interaction between human and murine cells or microenvironment or cytokines. Nevertheless, despite these limitations, murine models remain an invaluable tool for preclinical research, providing essential insights into CAR-T cells potential efficacy and safety before advancing to human clinical trials.

## 6 Conclusion

CAR-T cell therapy has revolutionized the cancer immunotherapy landscape. However, despite its remarkable successes in clinics in lymphoid malignancies, even if there are still some major challenges in myeloid malignancies, there are still challenges remaining as for solid tumors, including consistent antigen expression across all tumor cells, antigen loss or mutation, or other resistance mechanisms to immune cells. This review focused on the need to use robust 3D *in vitro* culture systems to study CAR-T therapy in solid tumors or in bone marrow microenvironment to develop CAR-T cell therapy for hematological malignancies such as acute myeloid leukemia.

2D co-culture systems provide useful initial insights into CAR-T cell biology, such as cytotoxicity, proliferation and cytokine production. The advantages regarding simplicity, low cost and real-time observation make 2D co-culture an indispensable tool for preliminary studies. However, their limitations in recapitulating the TME, including immunosuppressive elements, hypoxia, and extracellular matrix barriers reduce their predictive value for *in vivo* assays or clinical efficacy (109, 110). Thus, 3D culture systems have emerged as essential intermediate models to mimic some of the structural and biochemical complexities of tumors, enabling more accurate and rapid assessments of CAR-T cell infiltration, migration, killing capacity and exhaustion. Moreover, 3D models might be a suitable strategy to evaluate advanced designed CAR constructs; such as TRUCKs from the fourth generation, designed to secrete cytokines upon activation to modulate the TME, or CAR from the fifth generation. Several strategies to generate 3D spheroids and organoids have been developed. Direct cell aggregation techniques using low- or ultra-low-attachment plates are straightforward without any matrix. Matrix-based approaches incorporate extracellular matrix components and can be combined with microfluidic and organ-on-chip technologies, which allow precise control over dynamic parameters such as nutrient gradients and blood flow. Moreover, the use of patient-derived organotypic tumor models, incorporating components of the TME, provides a more accurate and physiologically relevant recapitulation of the TME for studying therapeutic responses.

The transition from 3D culture systems to *in vivo* murine models remain a critical step for preclinical validation. Murine models better capture the complexity of the disease model, tumor microenvironment, and tumor-immune cell dynamics compared to *in vitro* studies. However, the commonly used immunodeficient mice lack host immune cells and, therefore do not recapitulate tumor-immune interaction in absence of co-transplantation of PBMCs. Improvements and a combination of humanized mouse models and organ-on-chip technologies mimicking vascularized tumors may overcome differences between *in vitro* studies and clinical trials. However, despite all these advances, tumor immune escape mechanisms, antigen heterogeneity and the immunosuppressive TME remain limited in these models for assessment of CAR-T cell efficacy.

In conclusion, CAR-T cell therapy represents a pioneering approach to treat hematological malignancies, yet its success in solid tumors in clinical trials will require continued advancements in cell engineering, manufacturing, and modeling systems. Combining 2D and 3D culture systems with *in vivo* studies allows researchers to overview and evaluate CAR-T functionality. Ultimately, integrating these models with emerging technologies (microfluidic, bioprinting, organ-on-chip, etc.) might lead to more effective and durable CAR-T cell therapies for solid malignancies.

## Glossary

CAR-T: Chimeric Antigen Receptor T-cell
TME: Tumor Microenvironment
2D: Two-Dimensional
3D: Three-Dimensional
CAR: Chimeric Antigen Receptor
TIL: Tumor-Infiltrating T Lymphocyte
FDA: Food and Drug Administration
ALL: Acute Lymphoblastic Leukemia
LBCL: Large B-Cell Lymphoma
CLL: Chronic Lymphocytic Leukemia
MM: Multiple Myeloma
TRUCK: T-Cells Redirected for Universal Cytokine Killing
NSG: NOD Scid Gamma
PDX: Patient-Derived Xenograft
LDH: Lactate Dehydrogenase
ECM: Extracellular Matrix
CAF: Cancer-Associated Fibroblast
Treg: Regulatory T cell
PDAC: Primary Pancreatic Ductal Adenocarcinoma
EGF: Epidermal Growth Factor
bFGF: Basic Fibroblast Growth Factor
IGF: Insulin-Like Growth Factor
PEG: Polyethylene Glycol
PLGA: Poly-Lactic-Co-Glycolic Acid
VEGFR2: Vascular Endothelial Growth Factor Receptor 2
HER2: Human Epidermal Growth Factor Receptor 2
FRα: Folate Receptor Alpha
TAG-72: Tumor-Associated Glycoprotein 72
CD98hc: Cluster of Differenciation 98 Heavy Chain
B7-H3: B7 Homolog 3
HLA-A*02: Human Leukocyte Antigen A*02
CD19: Cluster of Differenciation 19
CD44: Cluster of Differenciation 44
EGFR: Epidermal Growth Factor Receptor
DCLK1: Doublecotrin-Like Kinase 1
CII: Collagenase II
MET: Mesenchymal-Epithelial Transition
NKG2D: Natural Killer Group 2 Member D
CD39: Cluster of Differenciation 39
CEA: Carcinoembryonic Antigen
CXCR3: C-X-C Chemokine Receptor Type 3
ROR1: Receptor Tyrosine Kinase-Like Orphan Receptor 1
TROP2: Trophoblast Cell Surface Antigen 2
EpCAM: Epithelial Cell Adhesion Molecule
IL13Rα2: Interleukin 13 Receptor Alpha 2
EphA3: Ephrin Type-A Receptor 3
P2X7: Purinergic Receptor P2X
CAIX: Carbonic Anhydrase IX

## References

[1] Steven MS Rosenberg A Packard BS Aebersold PM Diane Solomon MD Suzanne L. Topalian MD . Use of tumor-infiltrating lymphocytes and interleukin-2 in the immunotherapy of patients with metastatic melanoma. A preliminary Rep N Engl J Med. (1988) 319:1988. doi: 10.1056/NEJM198812223192527, PMID: 3264384

[2] Kwong MLM Yang JC . Lifileucel: FDA-approved T-cell therapy for melanoma. Oncologist. (2024) 2:648–50. doi: 10.1093/oncolo/oyae136, PMID: 39012213 PMC11299921

[3] Eshhar Z Waks T Gkoss G Schindler DG . Specific activation and targeting of cytotoxic lymphocytes through chimeric single chains consisting of antibody-binding domains and the γ or ζ subunits of the immunoglobulin and T-cell receptors. Proc Natl Acad Sci U. S. A. (1993) 90:720–4. doi: 10.1073/pnas.90.2.720, PMID: 8421711 PMC45737

[4] O’Leary MC Lu X Huang Y Lin X Mahmood I Przepiorka D . FDA Approval summary: Tisagenlecleucel for treatment of patients with relapsed or refractory b-cell precursor acute lymphoblastic leukemia. Clin Cancer Res. (2019) 25:1142–6. doi: 10.1158/1078-0432.CCR-18-2035, PMID: 30309857

[5] Wang Y Jain P Locke FL Maurer MJ Frank MJ Munoz JL . Brexucabtagene autoleucel for relapsed or refractory mantle cell lymphoma in standard-of-care practice: results from the US lymphoma CAR T consortium. J Clin Oncol. (2023) 41:2594–606. doi: 10.1200/JCO.22.01797, PMID: 36753699 PMC10489553

[6] Neelapu SS Locke FL Bartlett NL Lekakis LJ Miklos DB Jacobson CA . Axicabtagene ciloleucel CAR T-cell therapy in refractory large B-cell lymphoma. N Engl J Med. (2017) 377:2531–44. doi: 10.1056/nejmoa1707447, PMID: 29226797 PMC5882485

[7] Abramson JS Palomba ML Gordon LI Lunning MA Wang M Arnason J . Lisocabtagene maraleucel for patients with relapsed or refractory large B-cell lymphomas (TRANSCEND NHL 001): a multicentre seamless design study. Lancet. (2020) 396:839–52. doi: 10.1016/S0140-6736(20)31366-0, PMID: 32888407

[8] Raje N Berdeja J Lin Y Siegel D Jagannath S Madduri D . Anti-BCMA CAR T-cell therapy bb2121 in relapsed or refractory multiple myeloma. N Engl J Med. (2019) 380:1726–37. doi: 10.1056/nejmoa1817226, PMID: 31042825 PMC8202968

[9] Berdeja JG Madduri D Usmani SZ Jakubowiak A Agha M Cohen AD . Ciltacabtagene autoleucel, a B-cell maturation antigen-directed chimeric antigen receptor T-cell therapy in patients with relapsed or refractory multiple myeloma (CARTITUDE-1): a phase 1b/2 open-label study. Lancet vol. (2021) 398:314–24. doi: 10.1016/S0140-6736(21)00933-8, PMID: 34175021

[10] Tomasik J Jasiński M Basak GW . Next generations of CAR-T cells - new therapeutic opportunities in hematology? Front Immunol. (2022) 13:1034707. doi: 10.3389/fimmu.2022.1034707, PMID: 36389658 PMC9650233

[11] Ghorai SK Pearson AN . Current strategies to improve chimeric antigen receptor T (CAR-T) cell persistence. Cureus. (2024) 16. doi: 10.7759/cureus.65291, PMID: 39184661 PMC11343441

[12] Zhou X Tu S Wang C Huang R Deng L Song C . Phase I trial of fourth-generation anti-CD19 chimeric antigen receptor T cells against relapsed or refractory B cell non-hodgkin lymphomas. Front Immunol. (2020) 11:1–12. doi: 10.3389/fimmu.2020.564099, PMID: 33329526 PMC7731732

[13] Enblad G Karlsson H Gammelgård G Wenthe J Lövgren T Amini RM . A phase I / IIa trial using CD19-targeted third- generation CAR T cells for lymphoma and leukemia. Clin Cancer Res. (2013) 24:6185–94. doi: 10.1158/1078-0432.CCR-18-0426, PMID: 30097433

[14] Tang L Pan S Wei X Xu X Wei Q . Arming CAR-T cells with cytokines and more: Innovations in the fourth-generation CAR-T development. Mol Ther. (2023) 31:3146–62. doi: 10.1016/j.ymthe.2023.09.021, PMID: 37803832 PMC10638038

[15] Kagoya Y Tanaka S Guo T Anczurowski M Wang CH Saso K . A novel chimeric antigen receptor containing a JAK-STAT signaling domain mediates superior antitumor effects. Nat Med. (2018) 24:352–9. doi: 10.1038/nm.4478, PMID: 29400710 PMC5839992

[16] Pavlovic K Carmona-Luque MD Corsi GI Maldonado-Pérez N Molina-Estevez FJ Peralbo-Santaella E . Generating universal anti-CD19 CAR T cells with a de fi ned memory phenotype by CRISPR / Cas9 editing and safety evaluation of the transcriptome. Front Immunol. (2024) 15. doi: 10.3389/fimmu.2024.1401683, PMID: 38868778 PMC11167079

[17] Naeem M Abu H Bano N Ali Rashid Farooq M Abd Razak SI . Explorations of CRISPR / Cas9 for improving the long-term efficacy of universal CAR-T cells in tumor immunotherapy. Life Sci. (2023) 316:121409. doi: 10.1016/j.lfs.2023.121409, PMID: 36681183

[18] Tao R Han X Bai X Yu J . Revolutionizing cancer treatment : enhancing CAR-T cell therapy with CRISPR / Cas9 gene editing technology. Front Immunol. (2024) 15:1–11. doi: 10.3389/fimmu.2024.1354825, PMID: 38449862 PMC10914996

[19] Sakoda T Kasahara N Kedes L Ohyanagi M . Lentiviral vector - mediated gene transfer to endotherial cells compared with adenoviral and retroviral vectors lentiviral vector-mediated gene transfer to endotherial cells compared with. Prep Biochem Biotechnol. (2007) 6068:1–11. doi: 10.1080/10826060601039345, PMID: 17134978

[20] Vargas JE Chicaybam L Stein RT Tanuri A Cañedo AD Bonamino MH . Retroviral vectors and transposons for stable gene therapy : advances, current challenges and perspectives. J Transl Med. (2016) 14:1–15. doi: 10.1186/s12967-016-1047-x, PMID: 27729044 PMC5059932

[21] Logun M Colonna MB Mueller KP Ventarapragada D Rodier R Tondepu C . Label-free *in vitro* assays predict the potency of anti-disialoganglioside chimeric antigen receptor T-cell products. Cytotherapy. (2023) 25:670–82. doi: 10.1016/j.jcyt.2023.01.008.Logun, PMID: 36849306 PMC10159906

[22] Zhang Z Jiang D Yang H He Z Lui X Qin W . Modified CAR T cells targeting membrane-proximal epitope of mesothelin enhances the antitumor function against large solid tumor. Cell Death Dis. (2019) 10:1–12. doi: 10.1038/s41419-019-1711-1, PMID: 31209210 PMC6572851

[23] Michels KR Sheih A Hernandez SA Brandes AH Parrilla D Irwin B . Preclinical proof of concept for VivoVec, a lentiviral-based platform for *in vivo* CAR T-cell engineering. J Immunother. Cancer. (2023) 11:1–15. doi: 10.1136/jitc-2022-006292, PMID: 36918221 PMC10016276

[24] Jubelin C Munõz-Garcia J Griscom L Cochonneau D Olivier E Heymann MF . Three − dimensional *in vitro* culture models in oncology research. Cell Biosci. Front Immunol. (2022) 12:1–28. doi: 10.1186/s13578-022-00887-3, PMID: 36089610 PMC9465969

[25] Manduca N Maccafeo E De Maria R Sistigu A Musella M . 3D cancer models : One step closer to *in vitro* human studies. (2023), 1–18. doi: 10.3389/fimmu.2023.1175503, PMID: 37114038 PMC10126361

[26] Masoudnia MCM Verdurmen TVWPR . Organ-on-a-chip models for development of cancer immunotherapies, Cancer Immunol. Immunother. (3983) 72:3971. doi: 10.1007/s00262-023-03572-7, PMID: 37923890 PMC10700206

[27] Krysko DV Demuynck R Efimova I Naessens F Krysko O Catanzaro E . *In vitro* veritas : from 2D cultures to organ-on-a-chip models to study immunogenic cell death in the tumor microenvironment. Cells. (2022) 11. doi: 10.3390/cells11223705, PMID: 36429133 PMC9688238

[28] Nguyen DT Ogando-Rivas E Liu R Wang T Rubin J Jin L . CAR T cell locomotion in solid tumor microenvironment. Cells. (2022) 11:1–26. doi: 10.3390/cells11121974, PMID: 35741103 PMC9221866

[29] Marofi F Achmad H Bokov D Abdelbasset WK Alsadoon Z . Hurdles to breakthrough in CAR T cell therapy of solid tumors. Stem Cell Res Ther. (2022) 13:1–19. doi: 10.1186/s13287-022-02819-x, PMID: 35365241 PMC8974159

[30] Franchi-mendes T Eduardo R Domenici G . 3D cancer models : depicting cellular crosstalk within the tumour microenvironment. Cancers. (2021) 13:1–49. doi: 10.3390/cancers13184610, PMID: 34572836 PMC8468887

[31] Ehrmann GOGRL . The growth of cells on a transparent gel of reconstituted rat-tail collagen. J Natl Cancer Inst. (1956) 16:1375–403., PMID: 13320119

[32] Rheinwald HGJG . Serial cultivation of strains of human epidermal keratinocytes: the formation of keratinizing colonies from single cells. Cell. (1975) 6:331–43. doi: 10.1016/s0092-8674(75)80001-8, PMID: 1052771

[33] Moro LG Guarnier LP Azevedo MF Fracasso JAR Lucio MA de Castro MV . A brief review on the cell culture history: from harrison to organs-on-a-chip (2024). Available online at: https://www.mdpi.com/2073-4409/13/24/2068. 10.3390/cells13242068 PMC11674496 39768159

[34] Cacciamali A Villa R Dotti S . 3D cell cultures: evolution of an ancient tool for new applications. Front Physiol. (2022) 13:836480. doi: 10.3389/fphys.2022.836480, PMID: 35936888 PMC9353320

[35] Sakalem ME De Sibio MT da S. da Costa FA de Oliveira M . Historical evolution of spheroids and organoids, and possibilities of use in life sciences and medicine. Biotechnol J. (2021) 16. doi: 10.1002/biot.202000463, PMID: 33491924

[36] Corrò C Novellasdemunt L Li VSW . A brief history of organoids. Am J Physiol - Cell Physiol. (2020) 319:C151–65. doi: 10.1152/ajpcell.00120.2020, PMID: 32459504 PMC7468890

[37] Wiebel M Kailayangiri S Altvater B Meltzer J Grobe J Kupich S . Surface expression of the immunotherapeutic target GD2 in osteosarcoma depends on cell confluency. Cancer Rep. (2021) 4:1–9. doi: 10.1002/cnr2.1394, PMID: 33811471 PMC8551999

[38] McKenna MK Englisch A Brenner B Smith T Hoyos V Suzuki M . Mesenchymal stromal cell delivery of oncolytic immunotherapy improves CAR-T cell antitumor activity. Mol Ther. (2021) 29:1808–20. doi: 10.1016/j.ymthe.2021.02.004, PMID: 33571680 PMC8116608

[39] Köseer AS Loureiro LR Jureczek J Mitwasi N González Soto KE Aepler J . Validation of CD98hc as a therapeutic target for a combination of radiation and immunotherapies in head and neck squamous cell carcinoma. Cancers (Basel). (2022) 14. doi: 10.3390/cancers14071677, PMID: 35406454 PMC8997111

[40] Chuangchot N Jamjuntra P Yangngam S Luangwattananun P Thongchot S Junking M . Enhancement of PD-L1-attenuated CAR-T cell function through breast cancer-associated fibroblasts-derived IL-6 signaling via STAT3/AKT pathways. Breast Cancer Res. (2023) 25:1–16. doi: 10.1186/s13058-023-01684-7, PMID: 37480115 PMC10362675

[41] Wu H Xu Z Qi M Lui P Zhang B Wang Z . Interleukin-7 expression by CAR-T cells improves CAR-T cell survival and efficacy in chordoma. Cancer Immunol Immunother. (2024) 73:1–13. doi: 10.1007/s00262-024-03756-9, PMID: 39093440 PMC11297017

[42] Guo X Zheng H Luo W Zhang Q Liu J Yao K . 5T4-specific chimeric antigen receptor modification promotes the immune efficacy of cytokine-induced killer cells against nasopharyngeal carcinoma stem cell-like cells. Sci Rep. (2017) 7:1–13. doi: 10.1038/s41598-017-04756-9, PMID: 28687750 PMC5501797

[43] Englisch A Altvater B Kailayangiri S Hartmann W Rossig C . VEGFR2 as a target for CAR T cell therapy of Ewing sarcoma, Pediatr. Blood Cancer. (2020) 67:1–11. doi: 10.1002/pbc.28313, PMID: 32729251

[44] Luangwattananun P Junking M Sujjitjoon J Wutti-in Y Poungvarin N Thuwajit C . Fourth-generation chimeric antigen receptor T cells targeting folate receptor alpha antigen expressed on breast cancer cells for adoptive T cell therapy, Breast Cancer Res. Treat. (2021) 186:25–36. doi: 10.1007/s10549-020-06032-3, PMID: 33389403

[45] Suraiya AB Evtimov VJ Truong VX Boyd RL Forsythe JS Boyd NR . Micro-hydrogel injectables that deliver effective CAR-T immunotherapy against 3D solid tumor spheroids, Transl. Oncol. (2022) 24:101477. doi: 10.1016/j.tranon.2022.101477, PMID: 35905640 PMC9334344

[46] Schoutrop E Nilsson IM Hahn P Poiret T Kiessling R Wickström SL . Trogocytosis and fratricide killing impede MSLN-directed CAR T cell functionality. Oncoimmunology. (2022) 11. doi: 10.1080/2162402X.2022.2093426, PMID: 35898704 PMC9313125

[47] Zhang S Black G Kohli K Hayes BJ Miller C Koehne M . B7-H3 specific CAR T cells for the naturally occurring, spontaneous canine sarcoma model. Mol Cancer Ther. (2022) 21:999–1009. doi: 10.1158/1535-7163.MCT-21-0726, PMID: 35405743 PMC9381119

[48] Bassan D Weinberger L Yi J Kim T Weist MR Adams GB . HER2 and HLA-A*02 dual CAR-T cells utilize LOH in a NOT logic gate to address on-target off-tumor toxicity. J Immunother. Cancer. (2023) 11:1–12. doi: 10.1136/jitc-2023-007426, PMID: 38097342 PMC10729064

[49] McKenna MK Ozcan A Brenner D Watanabe N Legendre M Thomas DG . Novel banana lectin CAR-T cells to target pancreatic tumors and tumor-associated stroma. J Immunother. Cancer. (2023) 11:1–13. doi: 10.1136/jitc-2022-005891, PMID: 36653070 PMC9853244

[50] Rakhmatullina AR Zolotykh MA Filina YV Valiullina AK Zmievskaya EA Gafurbaeva DU . Multicellular cancer-stroma spheres (CSS) for *in vitro* assessment of CAR-T cell-associated toxicity. Cells. (2024) 13:1–14. doi: 10.3390/cells13221892, PMID: 39594640 PMC11593285

[51] de Oliveira KG Bång-Rudenstam A Beyer S Boukredine A Talbot H Governa V . Decoding of the surfaceome and endocytome in primary glioblastoma cells identifies potential target antigens in the hypoxic tumor niche. Acta Neuropathol. Commun. (2024) 12:1–20. doi: 10.1186/s40478-024-01740-z, PMID: 38414005 PMC10898066

[52] Reddy NR Maachi H Simic MS Yu W Tonai Y Cabanillas DA . Engineering synthetic suppressor T cells that execute locally targeted immunoprotective programs. Science. (2024) 386:eadl4793. doi: 10.1126/science.adl4793, PMID: 39636990 PMC11831968

[53] Witusik-Perkowska M Rieske P Hułas-Bigoszewska K Zakrzewska M Stawski R Kulczycka-Wojdala D . Glioblastoma-derived spheroid cultures as an experimental model for analysis of EGFR anomalies. J Neurooncol. (2011) 102:395–407. doi: 10.1007/s11060-010-0352-0, PMID: 20803305 PMC3089721

[54] Szöőr Á Tóth G Zsebik B Szabó V Eshhar Z Abken H . Trastuzumab derived HER2-specific CARs for the treatment of trastuzumab-resistant breast cancer: CAR T cells penetrate and eradicate tumors that are not accessible to antibodies. Cancer Lett. (2020) 484:1–8. doi: 10.1016/j.canlet.2020.04.008, PMID: 32289441

[55] Phanthaphol N Somboonpatarakun C Suwanchiwasiri K Chieochansin T Sujjitjoon J Wongkham S . Chimeric antigen receptor T cells targeting integrin αvβ6 expressed on cholangiocarcinoma cells. Front Oncol. (2021) 11:657868. doi: 10.3389/fonc.2021.657868, PMID: 33763382 PMC7982884

[56] Long C Li G Zhang C Joang T Li Y Duan X . B7-H3 as a target for CAR-T cell therapy in skull base chordoma. Front Oncol. (2021) 11:659662. doi: 10.3389/fonc.2021.659662, PMID: 34868903 PMC8634710

[57] Supimon K Sangsuwannukul T Sujjitjoon J Chieochansin T Junking M Yenchitsomanus Pt . Cytotoxic activity of anti-mucin 1 chimeric antigen receptor T cells expressing PD-1-CD28 switch receptor against cholangiocarcinoma cells. Cytotherapy. (2023) 25:148–61. doi: 10.1016/j.jcyt.2022.10.006, PMID: 36396553

[58] Zhou W Lei S Lui M Li D Huang Y Hu X . Injectable and photocurable CAR-T cell formulation enhances the anti-tumor activity to melanoma in mice. Biomaterials. (2022) 291:121872. doi: 10.1016/j.biomaterials.2022.121872, PMID: 36323072

[59] Galvagno F Leuci V Massa A Donini C Rotolo R Capellero S . Three-dimensional dynamics of mesothelin-targeted CAR.CIK lymphocytes against ovarian cancer peritoneal carcinomatosis. Cancer Immunol Immunother. (2024) 74:6. doi: 10.1007/s00262-024-03860-w, PMID: 39487859 PMC11531451

[60] Nagy L Mezősi-Csaplár M Rebenku I Vereb G Szöőr Á . Universal CAR T cells targeted to HER2 with a biotin-trastuzumab soluble linker penetrate spheroids and large tumor xenografts that are inherently resistant to trastuzumab mediated ADCC. Front Immunol. (2024) 15:1365172. doi: 10.3389/fimmu.2024.1365172, PMID: 38562932 PMC10982377

[61] Chiriaco C Donini C Cortese M Ughetto S Modica C Martinelli I . Efficacy of CAR-T immunotherapy in MET overexpressing tumors not eligible for anti-MET targeted therapy. J Exp Clin Cancer Res. (2022) 41:1–19. doi: 10.1186/s13046-022-02479-y, PMID: 36271379 PMC9585715

[62] Zou F Tan J Lui T Tang Y Zhang H Li J . The CD39+ HBV surface protein-targeted CAR-T and personalized tumor-reactive CD8+ T cells exhibit potent anti-HCC activity. Mol Ther. (2021) 29:1794–807. doi: 10.1016/j.ymthe.2021.01.021, PMID: 33484968 PMC8116602

[63] Li S Wang CS Montel-Hagen A Chen HC Lopez S Zhou O . Strength of CAR signaling determines T cell versus ILC differentiation from pluripotent stem cells. Cell Rep (2023) 42. doi: 10.1016/j.celrep.2023.112241.Strength, PMID: 36906850 PMC10315155

[64] Atik AF Suryadevara CM Schweller RM West JL Healy P Herndon Li JE . Hyaluronic acid based low viscosity hydrogel as a novel carrier for convection enhanced delivery of CAR T cells. J Clin Neurosci. (2019) 56:163–8. doi: 10.1016/j.jocn.2018.06.005.Hyaluronic, PMID: 30041899 PMC6185757

[65] Grosskopf AK Labanieh L Klysz DD Roth GA Xu P Adebowale O . Delivery of CAR-T cells in a transient injectable stimulatory hydrogel niche improves treatment of solid tumors. Sci Adv. (2022) 8:1–14. doi: 10.1126/sciadv.abn8264, PMID: 35394838 PMC8993118

[66] Jie J Mao D Cao J Feng P Yang P . Customized multifunctional peptide hydrogel scaffolds for CAR-T-cell rapid proliferation and solid tumor immunotherapy. ACS Appl Mater Interfaces. (2022) 14:37514–27. doi: 10.1021/acsami.2c10727, PMID: 35944246

[67] Wang K Chen Y Ahn S Zheng M Landoni E Dotti G . GD2-specific CAR T cells encapsulated in an injectable hydrogel control retinoblastoma and preserve vision. Nat Cancer. (2020) 1:990–7. doi: 10.1038/s43018-020-00119-y, PMID: 33898999 PMC8061756

[68] Hu Q Li H Archibong E Chen Q Ruan H Ahn H . Inhibition of post-surgery tumour recurrence via a hydrogel releasing CAR-T cells and anti-PDL1-conjugated platelets. Nat Biomed Eng. (2021) 5:1038–47. doi: 10.1038/s41551-021-00712-1, PMID: 33903744 PMC9102991

[69] Wang D Zhang M Qiu G Rong C Zhu X Qin G . Extracellular matrix viscosity reprogramming by in situ au bioreactor-boosted microwavegenetics disables tumor escape in CAR-T immunotherapy. ACS Nano. (2023) 17:5503–16. doi: 10.1021/acsnano.2c10845, PMID: 36917088

[70] Chao Y Wei T Li Q Liu B Hao Y Chen M . Metformin-containing hydrogel scaffold to augment CAR-T therapy against post-surgical solid tumors. Biomaterials. (2023) 295:122052. doi: 10.1016/j.biomaterials.2023.122052, PMID: 36827893

[71] Zhu C Ke L Ao X Chen Y Cheng H Xin H . Injectable supramolecular hydrogels for *in situ* programming of car-T cells toward solid tumor immunotherapy. Adv Mater. (2024) 36:1–15. doi: 10.1002/adma.202310078, PMID: 37947048

[72] Lizana-Vasquez GD Mendez-Vega J Cappabianca D Saha K Torres-Lugo M . *In vitro* encapsulation and expansion of T and CAR-T cells using 3D synthetic thermo-responsive matrices. RSC Adv. (2024) 14:13734–47. doi: 10.1039/d4ra01968g, PMID: 38681842 PMC11046447

[73] Lizana-Vasquez GD Ramasubramanian S Davarzani A Cappabianca D Saha K Karumbaiah L . *In vitro* assessment of thermo-responsive scaffold as a 3D synthetic matrix for CAR-T potency testing against glioblastoma spheroids. J Biomed Mater Res - Part A. (2024) 113:1–13. doi: 10.1002/jbm.a.37823, PMID: 39460647

[74] Liu X Zhao J Sho C Liu Z Shen H Dang J . Construction of CII-specific CAR-T to explore the cytokine cascades between cartilage-reactive T cells and chondrocytes. Front Immunol. (2020) 11:568741. doi: 10.3389/fimmu.2020.568741, PMID: 33343563 PMC7746615

[75] Joy JD Malacrida B Laforêts F Kotantaki P Maniati E Manchanda R . Human 3D ovarian cancer models reveal Malignant cell–intrinsic and –extrinsic factors that influence CAR T-cell activity. Cancer Res. (2024) 84:2432–49. doi: 10.1158/0008-5472.CAN-23-3007, PMID: 38819641 PMC11292204

[76] Zhang X Zhao Y Chen X . Collagen extracellular matrix promotes gastric cancer immune evasion by activating IL4I1-AHR signaling. Transl Oncol. (2024) 49:102113. doi: 10.1016/j.tranon.2024.102113, PMID: 39216468 PMC11402615

[77] Costa EC de Melo-Diogo D Moreira AF Carvalho MP Correia IJ . Spheroids formation on non-adhesive surfaces by liquid overlay technique: considerations and practical approaches. Biotechnol J. (2018) 13:1–12. doi: 10.1002/biot.201700417, PMID: 29058365

[78] Grandhi TSP Mebrahtu M Musso R Fullman A Nifong B Wisdom K . A microphysiological assay for studying T-cell chemotaxis, trafficking and tumor killing. Biofabrication. (2024) 17. doi: 10.1088/1758-5090/ad847f, PMID: 39378897

[79] Paterson K Paterson S Mulholland T Coffelt SB Zagnoni M . Assessment of CAR-T cell-mediated cytotoxicity in 3D microfluidic cancer co-culture models for combination therapy. IEEE Open J Eng. Med Biol. (2022) 3:86–95. doi: 10.1109/OJEMB.2022.3178302, PMID: 35813488 PMC9252335

[80] Wan Z Floryan MA Coughlin MF Zhang S Zhong AX Sheltin SE . New strategy for promoting vascularization in tumor spheroids in a microfluidic assay. Adv Healthc. Mater. (2023) 12:1–12. doi: 10.1002/adhm.202201784, PMID: 36333913 PMC10156888

[81] Chen Z Han S Sanny A Leung-Kwan Chan D Van Noort D Lim W . 3D hanging spheroid plate for high-throughput CAR T cell cytotoxicity assay. J Nanobiotechnology. (2022) 20:1–14. doi: 10.1186/s12951-021-01213-8, PMID: 35012567 PMC8744335

[82] Hirth E Cao W Peltonen M Kapetanovic E Dietsche C Svanberg S . Self-assembled and perfusable microvasculature-on-chip for modeling leukocyte trafficking. Lab Chip. (2023) 24:292–304. doi: 10.1039/d3lc00719g, PMID: 38086670 PMC10793075

[83] Luo Z Liu Z Liang Z Pan J Dong J . Injectable porous microchips with oxygen reservoirs and an immune-niche enhance the efficacy of CAR T cell therapy in solid tumors. ACS Appl Mater Interfaces. (2020) 12:56712–22. doi: 10.1021/acsami.0c15239, PMID: 33306365

[84] Martini S Drzeniek NM Stark R Reiner Kollert M Di W Reinke S . Long-term *in vitro* maintenance of plasma cells in a hydrogel-enclosed human bone marrow microphysiological 3D model system. Biofabrication. (2024) 16. doi: 10.1088/1758-5090/ad5dfe, PMID: 38955197

[85] Maulana TI Teufel C Cipriano M Hudecek M Alb M Loskull P . Breast cancer-on-chip for patient-specific efficacy and safety testing of CAR-T cells. Cell Stem Cell. (2024) 31:989–1002.e9. doi: 10.1016/j.stem.2024.04.018, PMID: 38754430

[86] Cho Y Laird MS Bishop T Li R Jazwinska DE Ruffo E . CAR T cell infiltration and cytotoxic killing within the core of 3D breast cancer spheroids under the control of antigen sensing in microwell arrays. APL Bioeng. (2024) 8. doi: 10.1063/5.0207941, PMID: 39049849 PMC11268919

[87] Tang M Qu Y He P Yao E Guo T Yu D . Heat-inducible CAR-T overcomes adverse mechanical tumor microenvironment in a 3D bioprinted glioblastoma model. Mater Today Bio. (2024) 26. doi: 10.1016/j.mtbio.2024.101077, PMID: 38765247 PMC11099333

[88] Önder CE Moustafa-Oglou M Schröder SM Hartkopf AD Koch A Seitz CM . Precision immunotherapy utilizing adapter CAR-T cells (AdCAR-T) in metastatic breast cancer leads to target specific lysis. Cancers (Basel). (2024) 16:221–8. doi: 10.3390/cancers16010168, PMID: 38201595 PMC10778501

[89] Jiang Y Sun X Song X Li Z Zhang W . Patient-derived bladder cancer organoid model to predict sensitivity and feasibility of tailored precision therapy. Curr Urol. (2023) 17:221–8. doi: 10.1097/CU9.0000000000000219, PMID: 37994334 PMC10662868

[90] Ventin M Cattaneo G Arya S Jia J Gelmi MC Sun Y . Chimeric antigen receptor T cell with an inducible caspase-9 suicide gene eradicates uveal melanoma liver metastases via B7-H3 targeting. Clin Cancer Res. (2024) 30:3243–58. doi: 10.1158/1078-0432.CCR-24-0071, PMID: 38767611 PMC11572477

[91] Martins P D'Souza RCJ Skarne N Lekieffre L Horsefield S Ranjankumar M . EphA3 CAR T cells are effective against glioblastoma in preclinical models. J Immunother. Cancer. (2024) 12. doi: 10.1136/jitc-2024-009403, PMID: 39111832 PMC11308892

[92] Bandara V Niktaras VM Willet VJ Chapman H Likman NA Macpherson AM . Engineered CAR-T cells targeting the non-functional P2X purinoceptor 7 (P2X7) receptor as a novel treatment for ovarian cancer. Clin Transl Immunol. (2024) 13:1–17. doi: 10.1002/cti2.1512, PMID: 38800555 PMC11116765

[93] Marc Wehrli MVM Guinn S Birocchi F Kuo A Sun Yi Larson RC . Mesothelin CAR T-cells secreting anti-FAP/anti-CD3 molecules efficiently target pancreatic adenocarcinoma and its stroma. Clin Cancer Res. (2024) 30:1859–77. doi: 10.1158/1078-0432.CCR-23-3841, PMID: 38393682 PMC11062832

[94] Logun M Wang X Sun Y Ming G Song H Rourke DMO . Clinical and Translational Report Patient-derived glioblastoma organoids as real-time avatars for assessing responses to clinical CAR-T cell therapy Clinical and Translational Report Patient-derived glioblastoma organoids as real-time avatars for assessin. Stem Cell. (2025) 32:1–10. doi: 10.1016/j.stem.2024.11.010, PMID: 39657679 PMC11808387

[95] Wang Y Buck A Piel B Zerefa L Murugan N Coherd CD . Affinity fine-tuning anti-CAIX CAR-T cells mitigate on-target off-tumor side effects. Mol Cancer. (2024) 23:1–16. doi: 10.1186/s12943-024-01952-w, PMID: 38491381 PMC10943873

[96] Sureban SM Berahovich R Zhou H Xu S Wy L Ding K . DCLK1 monoclonal antibody-based CAR-T cells as a novel treatment strategy against human colorectal cancers. Cancers (2019) 12:1–17. doi: 10.3390/cancers12010054, PMID: 31878090 PMC7016951

[97] Baysoy A Bai Z Satija R Fan R . The technological landscape and applications of single-cell multi-omics. Nat Rev Mol Cell Biol. (2023) 24:695–713. doi: 10.1038/s41580-023-00615-w, PMID: 37280296 PMC10242609

[98] Alsalloum A Alrhmoun S Perik-Zavosdkaia O Fisher M Volynetd M Lopatnikova J . Decoding NY-ESO-1 TCR T cells : transcriptomic insights reveal dual mechanisms of tumor targeting in a melanoma murine xenograft model, no. November. (2024) 15:1–11. doi: 10.3389/fimmu.2024.1507218, PMID: 39660132 PMC11628372

[99] Qu N Meng Q Bi M Liu H Cao X . Low-intensity pulsed ultrasound combined with microbubble mediated JNK/c-Jun pathway to reverse multidrug resistance in triple-negative breast cancer. Sci Rep. (2024) 14:27250. doi: 10.1038/s41598-024-78272-y, PMID: 39516537 PMC11549295

[100] Meher N Bidkar AP Wadhwa A Bobba KN Dhrona S Dasari C . PET imaging using 89 zr-labeled starPEG nanocarriers reveals heterogeneous enhanced permeability and retention in prostate cancer. Mol Cancer Ther. (2025) 24:1–11. doi: 10.1158/1535-7163.MCT-24-0024, PMID: 39331510 PMC11694059

[101] Xiao W Xu L Wang J Yu K Xu B Que Y . FGFR4-specific CAR-T cells with inducible caspase-9 suicide gene as an approach to treat rhabdomyosarcoma. Cancer Gene Ther. (2024) 31:1571–84. doi: 10.1038/s41417-024-00823-2, PMID: 39183354 PMC11489081

[102] Glez-Vaz J Azpilikueta A Olivera I Cirella A Teijeira A Ochoa MC . Soluble CD137 as a dynamic biomarker to monitor agonist CD137 immunotherapies. J Immunother. Cancer. (2022) 10:1–14. doi: 10.1136/jitc-2021-003532, PMID: 35236742 PMC8896037

[103] Kellermayer Z Tahri S E je Jong MM Papazian N Fonkema C Stoetman ECG . Interferon gamma - mediated prevention of tumor progression in a mouse model of multiple myeloma, no. December. (2023) 8:1–16. doi: 10.1002/hem3.70047, PMID: 39624831 PMC11609740

[104] Rubino V Hüppi M Höpner S Tortola L Schnüriger N Legenne H . IL-21/IL-21R signaling renders acute myeloid leukemia stem cells more susceptible to cytarabine treatment and CAR T cell therapy. Cell Rep Med. (2024) 5. doi: 10.1016/j.xcrm.2024.101826, PMID: 39536753 PMC11604404

[105] Park HB Hyun Kim K Hwan Kim J Il Kim S Mi Oh Y Kang Miseung . Improved safety of chimeric antigen receptor T cells indirectly targeting antigens via switchable adapters. Nat Commun. (2024) 15. doi: 10.1038/s41467-024-53996-7, PMID: 39557825 PMC11574259

[106] Cirstoiu-Hapca A Buchegger F Lange N Bossy L Gurny R Delie F . Benefit of anti-HER2-coated paclitaxel-loaded immuno-nanoparticles in the treatment of disseminated ovarian cancer: Therapeutic efficacy and biodistribution in mice. J Control. Release. (2010) 144:324–31. doi: 10.1016/j.jconrel.2010.02.026, PMID: 20219607

[107] Kurniawan A Mahendra I Febrian MB Utama MS Gunadi WJ Wahyudianingsih R . Biological evaluation of hydroxyapatite zirconium nanoparticle as a potential radiosensitizer for lung cancer X-ray induced photodynamic therapy. Appl Radiat. Isot. (2024) 217:111615. doi: 10.1016/j.apradiso.2024.111615, PMID: 39632318

[108] Annie L Hsieh BLS Ganesh S Kula T Irshad M Ferenczi EA Wang W . Widespread neuroanatomical integration and distinct electrophysiological properties of glioma-innervating neurons. Proc Natl Acad Sci U. S. A. (2024) 121. doi: 10.1073/pnas.2417420121, PMID: 39630872 PMC11648874

[109] Biosensors C Edmondson R Broglie JJ Adcock AF Yang L . Three-dimensional cell culture systems and their applications in drug discovery and cell-based biosensors. Assay Drug Dev Technol. (2014) 12:207–18. doi: 10.1089/adt.2014.573, PMID: 24831787 PMC4026212

[110] Clémence D Robin D Pierre D Corinne A . Development and cytotoxic response of two proliferative MDA- culture models of triple-negative basal-like breast cancer cell lines. Oncotarget. (2017) 8:95316–31. doi: 10.18632/oncotarget.20517, PMID: 29221130 PMC5707024

