# Correction: From spheroids to organoids: next-generation models for CAR-T cell therapy research in solid tumors

**DOI:** 10.3389/fimmu.2025.1738434

**Published:** 2025-12-02

**Authors:** Mégane Jassin, Alix Block, Laury Désiront, Louise Vrancken, Céline Grégoire, Frédéric Baron, Grégory Ehx, Thi Tham Nguyen, Jo Caers

**Affiliations:** 1 Laboratory of Hematology, Interdisciplinary Cluster for Applied Genoproteomics Institute (GIGA) Institute, University of Liege, Liege, Belgium; 2 Department of Hematology, University Hospital of Liege, Liege, Belgium; 3 Walloon Excellence in Life Sciences and Biotechnology (WELBIO) Department, Walloon Excellenxe in Life Research Institute, Wavre, Belgium

**Keywords:** car-t, chimeric antigen receptor T cells, solid tumor, 3D culture, tumor microenvironment, spheroid, organoid, immunotherapy

There was a mistake in the caption of **Figure 6** as published. Figure uncomplete (missing words, empty square without “collagen matrix, tumor organoid, hydrogel matrix, biopsy derived cells”). The corrected caption of **Figure 6** appears below.

**Figure 6:**
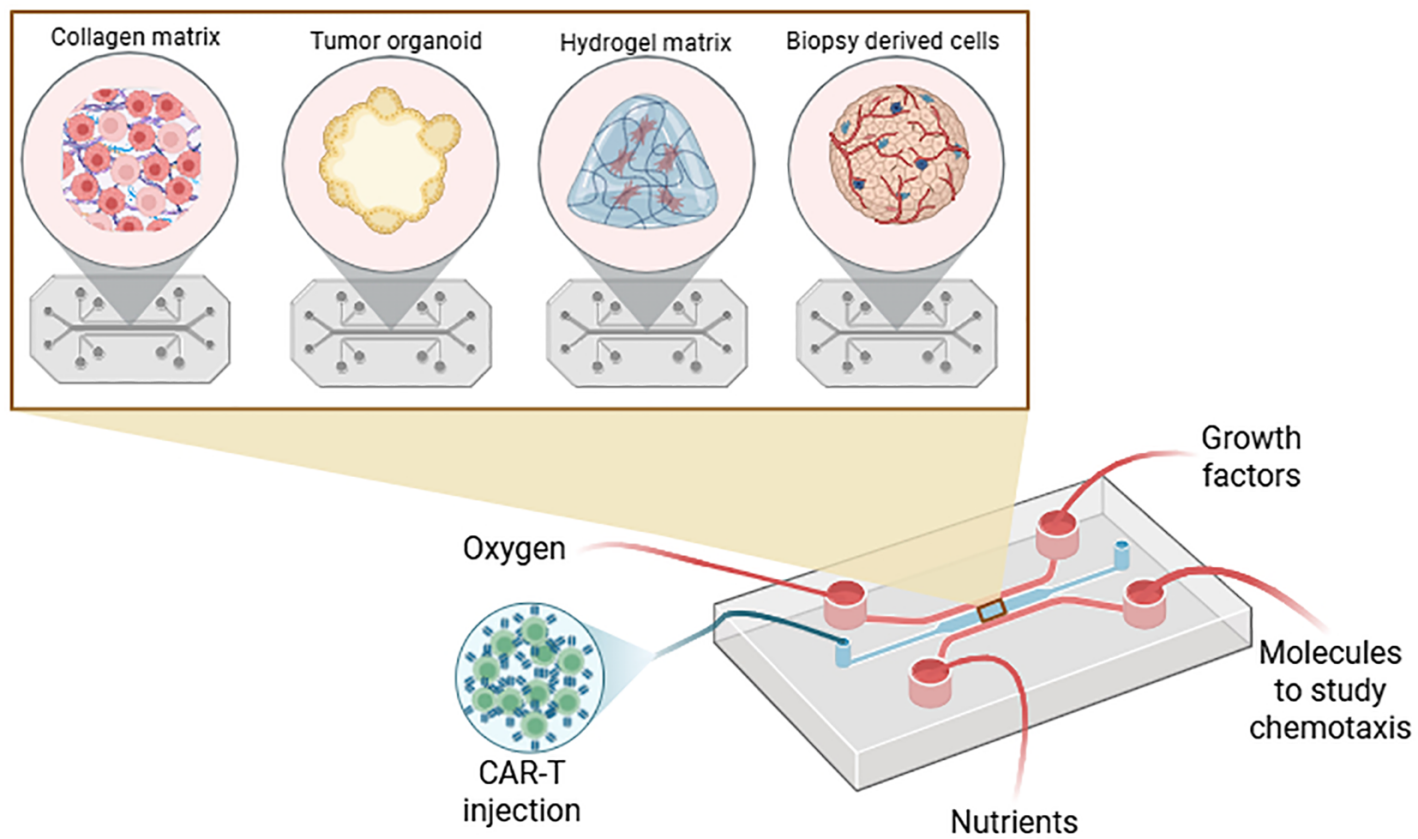
Schematic representation of using matrix coupled with microfluidic, organ-on-chip and bioprinting. Microfluidic device is composed of different channels, one to culture the spheroid or organoid with CAR-T cells while other channels can bring oxygen, nutrients, chemotaxis molecules or growth factors or can mimick blood vessels. Channels can be coupled with filters to remove debris and dead cells. This figure was generated on Biorender.

The original version of this article has been updated.

